# Implications of early and guideline adherent physical therapy for low back pain on utilization and costs

**DOI:** 10.1186/s12913-015-0830-3

**Published:** 2015-04-09

**Authors:** John D Childs, Julie M Fritz, Samuel S Wu, Timothy W Flynn, Robert S Wainner, Eric K Robertson, Forest S Kim, Steven Z George

**Affiliations:** Army Medical Department Center and School, US Army-Baylor University Doctoral Program in Physical Therapy, 3151 Scott Rd., Rm. 2307, JBSA Fort Sam Houston, San Antonio, TX 78234 USA; Department of Physical Therapy, University of Utah, 520 Wakara Way, Salt Lake City, UT 84108 USA; Department of Health Services Research, Management and Policy, College of Public Health and Health Professions, University of Florida, 1329 SW 16th St., Rm. 5231, Gainesville, FL 32610-0177 USA; EIM School of Physical Therapy, South College, 3904 Lonas Dr, Knoxville, TN 37909 USA; Doctor of Physical Therapy Program, University of Texas at El Paso, 500 W. University Avenue, El Paso, TX 79968 USA; US Army Medical Department Center and School, US Army-Baylor MHA/MBA Program, 3599 Winfield Scott Rd., Bldg. 2841, JBSA Fort Sam Houston, San Antonio, TX 78234-6135 USA; Department of Physical Therapy, Director, Brooks-PHHP Research Collaboration, University of Florida, P.O. Box 100154, Gainesville, FL 32610-0154 USA

**Keywords:** Guideline adherence, Low back pain, Physical therapy, Timing, Utilization and costs

## Abstract

**Background:**

Initial management decisions following a new episode of low back pain (LBP) are thought to have profound implications for health care utilization and costs. The purpose of this study was to evaluate the impact of early and guideline adherent physical therapy for low back pain on utilization and costs within the Military Health System (MHS).

**Methods:**

Patients presenting to a primary care setting with a new complaint of LBP from January 1, 2007 to December 31, 2009 were identified from the MHS Management Analysis and Reporting Tool. Descriptive statistics, utilization, and costs were examined on the basis of timing of referral to physical therapy and adherence to practice guidelines over a 2-year period. Utilization outcomes (advanced imaging, lumbar injections or surgery, and opioid use) were compared using adjusted odds ratios with 99% confidence intervals. Total LBP-related health care costs over the 2-year follow-up were compared using linear regression models.

**Results:**

753,450 eligible patients with a primary care visit for LBP between 18–60 years of age were considered. Physical therapy was utilized by 16.3% (n = 122,723) of patients, with 24.0% (n = 17,175) of those receiving early physical therapy that was adherent to recommendations for active treatment. Early referral to guideline adherent physical therapy was associated with significantly lower utilization for all outcomes and 60% lower total LBP-related costs.

**Conclusions:**

The potential for cost savings in the MHS from early guideline adherent physical therapy may be substantial. These results also extend the findings from similar studies in civilian settings by demonstrating an association between early guideline adherent care and utilization and costs in a single payer health system. Future research is necessary to examine which patients with LBP benefit early physical therapy and determine strategies for providing early guideline adherent care.

**Electronic supplementary material:**

The online version of this article (doi:10.1186/s12913-015-0830-3) contains supplementary material, which is available to authorized users.

## Background

Low back pain (LBP) is among the most common reasons to visit a physician and up to 25% of Americans report an incidence of back pain within the previous three months [1]. Combined direct and indirect costs for LBP are reported to be between $85 billion and $238 billion, and expenditures for back pain are rising more quickly than overall health expenditures [1-3]. While the vast majority of LBP episodes resolve within 2–4 weeks, 25% of patients will experience recurrent episodes within one year and the prevalence of chronic LBP has been reported to be on the rise [4,5].

The Military Health System (MHS) is responsible for providing health care to 10 million active duty and retired military personnel and their dependents, representing one of the largest single payer health systems in the United States. Prevalence estimates for LBP within the MHS are similar to the rest of society [6]. High costs of treatment for LBP and its impact on readiness of active duty members make LBP a particular concern for the MHS. LBP is the leading cause of medical discharge across all military services [7] and has been associated with high rates of medical evacuation from deployment [8]. Back pain in active duty members, as in civilian populations, is often accompanied by psychological distress, [9] increasing the risk for persistent pain and disability. Opioid medications are also frequently overused as an initial strategy for managing pain conditions such as LBP, a particular concern for the MHS, [10]. The need to improve the management of patients with pain has become a priority for the MHS [11].

Recommendations in clinical guidelines for acute, non specific LBP in both military and civilian settings are to avoid opioids as a first-line medication and avoid advanced imaging procedures such as MRI or CT scan [12-16]. However, research conducted mostly in civilian settings demonstrate clinical practice remains inconsistent with these recommendations, [17] with excess use of unendorsed care early in the care process contributing to the high costs of managing LBP, adverse events, and increasing risk of chronicity [4,17]. Several guidelines suggest a delay in referral to physical therapy for 2–4 weeks to allow for spontaneous recovery, [12,15,16] but emerging research with civilian populations has found cost savings when referrals to physical therapy occur early in the care process for patients with acute LBP symptoms, particularly if the physical therapy care provided focuses on active treatment approaches [18,19].

Further research is needed to delineate optimal LBP care pathways for comprehensive single payer settings such as the MHS that could be a model for other health care delivery systems and payment models [20]. Therefore, our goal in this study was to evaluate the impact of both the timing and adherence of physical therapy for individuals with LBP within the MHS alone and in combination on health care costs and utilization of advanced imaging, spine injections, surgery, or opioid use. We also sought to determine if the results seen in civilian payer environments with respect to timing and adherence would be observed in the MHS, and given the robust size of the MHS claims database, evaluate the interactions of these factors on outcomes. The issue of optimal care pathways for costly conditions in single payer systems is especially important given that the potential direction of pending health care reform favors consolidation in the United States [21].

## Methods

### Description of the data source

The data source for this project was the MHS Management Analysis and Reporting Tool (M2), which is maintained and operated by the Tricare Management Activity. M2 is an ad hoc application that tracks health care utilization across the MHS in support of health operations. M2 links claims and demographic data to provide summary and detailed views of population, clinical, and financial health utilization data. The database is updated monthly via an electronic feed from each of the Military Treatment Facility (MTF) regions worldwide and currently reflects the combined experience of more than 9 million MHS beneficiary members. M2 includes data from both the direct care system (care provided in MTFs) and commercial network claims (care provided to MHS beneficiaries at civilian facilities), thus these databases were merged for the purposes of this analysis. Prior to being transferred to the investigators, all traceable person-specific identifying factors was transformed into anonymous, coded study numbers to protect subjects’ privacy. The study protocol was approved by the 81^st^ Medical Group (Keesler Air Force Base, Biloxi, MS) and University of Florida Institutional Review Boards. A data sharing agreement for de-identified data was obtained from the Tricare Management Activity.

### Identification of study sample

Patients in the MHS with a new consultation to a primary care provider for standard diagnosis of LBP from January 1, 2007 to December 31, 2009 were identified. The date of new consultation was defined as the primary care index date. Patients had to be continuously eligible in the MHS database for 12 months prior to and 24 months following the index date to be included. A new consultation required that no care related to LBP was included in the MHS for 12 months prior to the index date. LBP diagnoses were identified through standard ICD-9 codes (Additional file 1). Only the first eligible index date for a patient was included, ensuring a patient was included only once in the study sample. Primary care providers were defined as Family Practice, Internal Medicine, or Flight Medicine providers. Other eligibility criteria included age between 18–60 on the index date, no co-morbid diagnosis of possible non-musculoskeletal sources of LBP (e.g., kidney stones, urinary tract infection, etc.) within 4 weeks of the index date (Additional file 1), no prior history of spine surgery or spine trauma based on related current procedural terminology (CPT)-4 codes at any time prior to the index date.

### Covariate variables

We recorded age, gender, marital status, race, rank, and geographic region across 12 different geographic regions around the world. Visits at a military treatment facility (MTF) versus “purchased” care that occurred outside of a MTF but reimbursed by the MHS via TRICARE®, military service (Army, Navy, etc.), beneficiary status (active duty, retired, etc.), and use of opioid medication were recorded. We also recorded co-morbid conditions that might influence LBP prognosis, including mental health (depression, anxiety disorder, bipolar, schizophrenia, or other psychotic disorders), neck/thoracic pain, or fibromyalgia diagnoses by identifying the relevant ICD-9 codes over 12-months prior to the index date (Additional file 1). The data set did not contain clinical data on symptom duration/location/severity, physical examination findings, potential psychosocial variables, or patient-centered clinical outcomes (ie. pain, function, disability, patient satisfaction, etc.).

### Physical therapy utilization

We considered the 90-day period following the primary care index date to determine details about physical therapy utilization. If a physical therapy visit occurred with a LBP-related ICD-9 code during this period, the patient was defined as utilizing physical therapy. Within that time-frame, patients who were received physical therapy within 14 days of the index date were defined as having received early physical therapy. The cut point of 14 days was based on the threshold used in previous studies to classify early physical therapy [18]. Those patients receiving physical therapy between 14 and 90 days from the index date were defined as receiving delayed physical therapy.

For patients utilizing physical therapy, the content of physical therapy visits received during the physical therapy episode of care was examined. An episode of care was defined as the number of days between initial and final physical therapy visits. The episode of care was considered complete once no additional physical therapy visit occurred within 30 consecutive days of the last visit. If only 1 physical therapy visit was received, the patient was not included in the analysis of adherence because these patients did not have an adequate number of visits with which to judge the content of the episode of care. CPT codes from each visit were used to categorize the content of physical therapy as adherent or non-adherent to the evidence-based recommendation for active physical therapy using a procedure described in detail elsewhere [18]. Active codes included CPT codes for therapeutic exercise or neuromuscular reeducation, for example, while passive codes included those codes for passive modalities including hot packs and ultrasound. A third category of allowed codes was used for codes that are neither active nor passive such as evaluations, and equipment-based codes. Numbers of active and passive codes were totaled for visits during the first 14 days of the episode of care (phase 1); and beyond 14 days (phase 2). Manual therapy was allowed (not categorized as active or passive) if it occurred during Phase 1 of the episode of care and passive if it occurred during Phase II of the episode of care based on evidence indicating the benefit of manual therapy when provided early in episodes of care [22]. For each treatment, the ratio of active codes utilized was calculated (number of active codes / (number of active codes + number of passive codes) × 100%). An episode of care was categorized as adherent when greater than 75% of the codes utilized during Phase I and Phase II were codes designating active or allowed physical therapy interventions and at least one active code was utilized during each session. Episodes of care not meeting these criteria were categorized as non-adherent.

### Outcome variables

We examined a 24-month period that began at the index date to observe health care utilization and cost outcomes. The following utilization outcomes were recorded when occurring with a LBP-related ICD-9 code: additional physician visits, advanced imaging (magnetic resonance imaging (MRI) or computed tomography (CT), spinal injections, spine surgery (discectomy, fusion, rhizotomy, or laminectomy), emergency department visits, prescription medication use, and opioid medication use. Costs related to these procedures were also recorded and summed to demonstrate Total LBP-related costs. Non-LBP healthcare costs for the 24-month period were also recorded and total healthcare costs were calculated.

### Data analysis

Descriptive statistics were calculated. Multivariate logistical regression using all covariates as potential predictors was performed to determine factors associated with physical therapy utilization. For patients utilizing physical therapy, descriptive statistics for costs (total LBP-related costs and total non-LBP costs) and utilization were further examined on the basis of timing (early vs. delayed) and content (adherent vs. non-adherent). Additionally, combined categories of timing and adherence were examined (early + adherent, delayed + non-adherent, etc.). Utilization outcomes were compared using adjusted odds ratios (AOR) with 99% confidence intervals derived from logistic regression controlling for all demographic and baseline co-morbid characteristics. Similarly, multivariate linear regression was performed to examine the relationship between log transformation of total and/or LBP-related healthcare costs and physical therapy utilization adjusting for all covariates.

## Results

**Figure 1:**
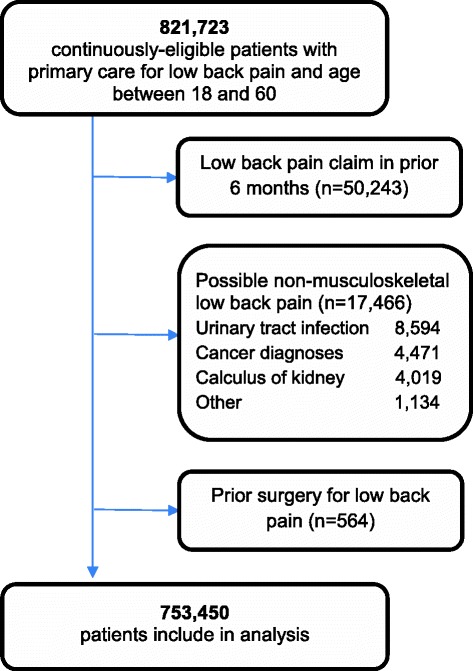
**Subject inclusion flow chart.**

821,723 continuously-eligible patients with a primary care visit for LBP between the ages of 18 and 60 at the index visit were considered for inclusion. Of these, we excluded 50,243 (6.1%) who received care with a LBP-related ICD-9 code within the prior 12 months, 17,466 (2.1%) with a possible non-musculoskeletal source of LBP, and 564 (0.07%) with prior surgery for LBP 753,450 unique patients were included in the analysis (Figure 1). Mean age was 36.9 years (sd = 12.5), with 46.8% being female. Among all patients 34.2% had a history of opioid use within the 12 months prior to the index visit for a non LBP-related complaint. Baseline demographic characteristics are provided in Table 1.

**Table 1:** **Demographics and baseline co-morbid characteristics for entire sample and based on physical therapy utilization**

		**All patients (n = 753,450)**	**PT user (n = 122,723)**	**No PT Referral (n = 630,727)**	**Physical therapy timing (n = 122,723)**		**Physical therapy adherence (n = 71,559)**	
					**Early (n = 72,641)**	**Delayed (n = 50,082)**	**Adherent (n = 30,917)**	**Non-adherent (n = 40,642)**
Age		36.9 (12.5)	35.0 (11.8)	37.2 (12.6)	34.4 (11.9)	35.9 (11.6)	36.2 (12.2)	37.7 (12.4)
Gender (% female)		46.8%	42.2%	47.7%	41.8%	42.8%	45.6%	52.0%
Index visit co‐payment		$6.26 (26.5)	$3.25 (14.4)	$6.85 (28.2)	$3.51 (13.7)	$2.86 (15.3)	$4.37 (15.9)	$6.02 (18.6)
*Common beneficiary category*								
	Dependents of active duty or guard/reserve on active duty	19.5%	16.2%	20.2%	15.3%	21.2%	17.9%	21.8%
	Retired	14.6%	9.6%	15.5%	8.6%	15.2%	12.0%	13.5%
	All others	21.3%	14.6%	22.6%	14.5%	22.6%	18.1%	22.0%
	Active duty and guard/reserve on active duty	44.6%	59.7%	41.7%	61.7%	41.%	52.0%	42.6%
Number of LBP diagnosis codes		1.5 (1.5)	1.4 (1.3)	1.5 (1.6)	1.5 (1.3)	1.3 (1.2)	1.5 (1.3)	1.8 (1.7)
Number of prescription medications		14.2 (10.5)	14.5 (10.6)	14.2 (10.5)	13.7 (10.4)	15.7 (10.8)	14.9 (10.7)	15.1 (10.8)
Co‐morbid mental health condition		17.%	15.6%	17.2%	15.3%	16.1%	16.3%	17.0%
Co‐morbid fibromyalgia diagnosis		2.9%	2.9%	2.9%	3.2%	2.4%	2.9%	3.8%
Co‐morbid neck/thoracic spine condition		9.1%	11.%	8.7%	12.7%	8.6%	10.2%	12.9%
Opioid use prior to index visit		34.2%	34.6%	34.1%	34.9%	34.2%	35.0%	36.0%
Hospitalization prior to index visit		7.2%	7.3%	7.2%	7.2%	7.4%	7.8%	7.7%
Total medical costs prior to index visit		$3515.62 (7357.68)	$3801.43 (8453.37)	$3459.24 (7120.32)	$3838.39 (9057.89)	$3747.74 (7488.64)	$3915.49 (9512.67)	$3801.73 (7407.62)

Values represent mean (standard deviation) unless otherwise indicated.

### Physical therapy utilization

Physical therapy was utilized within 90 days of index visit by 16.3% (n = 122,723) of patients. The mean number of physical therapy visits in the episode of care was 7.1 (sd = 12.2). Median time to physical therapy among all patients utilizing physical therapy was 9 days (interquartile range: 0, 27). Among patients who utilized physical therapy, 59.2% (n = 72,641) were categorized as receiving early physical therapy and 40.8% (n = 50,082) received delayed physical therapy. Patients who received early physical therapy had a mean of 7.3 (sd = 12.9) physical therapy visits compared to 6.8 (sd = 11.0) among patients receiving delayed physical therapy (Table 2).

**Table 2:** **Utilization and costs outcomes occurring over the 2-year follow-up period for the entire sample and based on the timing and adherence of physical therapy**

		**All patients (n = 753,450)**	**PT users (n = 122,723)**	**No PT Referral (n = 630,727)**	**Physical therapy timing (n = 122,723)**		**Physical therapy adherence (n = 71,559)**	
					**Early (n = 72,641)**	**Delayed (n = 50,082)**	**Adherent (n = 30,917)**	**Non-Adherent (n = 40,642)**
**Utilization outcomes**								
	Number of physical therapy visits (mean, sd)	1.2 (5.6)	7.1 (12.2)	0.0 (0.0)	7.3 (12.9)	6.8 (11.0)	6.2 (7.6)	15.0 (17.2)
	Advanced imaging	11.7%	15.6%	11.0%	11.9%	21.0%	17.0%	22.7%
	Lumbar spinal injections	6.5%	11.1%	5.7%	8.7%	14.6%	11.7%	13.8%
	Lumbar spine surgery	1.4%	2.5%	1.2%	1.9%	3.2%	2.6%	3.0%
	Opioid medication use	62.9%	64.0%	62.7%	59.7%	70.3%	65.2%	66.0%
**Costs incurred over the 24 month period** (mean, standard error)								
	Prescription medications	$1158.15 (4.45)	$768.26 (9.19)	$1,234.44 (5.01)	$772.20 (13.00)	$762.74 (12.44)	$886.27 (19.82)	$1233.90 (19.86)
	Inpatient costs	$10,958.82 (61.51)	$11,816.72 (156.74)	$10,780.45 (66.76)	$11,089.39 (196.72)	$12,840.89 (255.97)	$11,639.95 (300.06)	$11,292.57 (249.15)
	Total LBP costs	$1,217.80 (4.02)	$2,318.88 (14.24)	$1,003.56 (3.86)	$1,828.24 (15.28)	$3,030.53 (26.64)	$2,426.88 (30.04)	$2,733.57 (26.92)
	Non‐LBP healthcare costs	$8,281.37 (17.36)	$9,099.91 (45.24)	$8,122.11 (18.77)	$8,687.25 (59.52)	$9,698.47 (69.46)	$9,285.09 (89.76)	$9,157.06 (74.71)

### Healthcare utilization and costs based on timing

Utilization of specific services over the 2-year follow-up period is outlined in Table 2. Table 3 presents adjusted odds ratios (AOR) for the utilization outcomes based on the timing of Physical Therapy, which were obtained from logistic regression models that adjusted all demographic and baseline co-morbid characteristics listed in Table 1. Patients who received early physical therapy had a decreased likelihood of receiving advanced imaging (AOR = 0.52, 99% CI: 0.50, 0.54), spinal injections (AOR = 0.56, 99% CI: 0.53, 0.59), lumbar spine surgery (AOR = 0.59, 99% CI: 0.54, 0.65), or opioid use (AOR = 0.62, 99% CI: 0.60, 0.64). Total LBP-related costs during the 2-year follow-up for patients receiving early physical therapy were an average $1202.29 lower (95% CI: 1142.09-1262.49) compared to patients receiving delayed physical therapy, with a mean cost of $1,828.24 (SE = 15.28) and $3,030.53 (SE = 26.64), respectively. Similarly, non-LBP healthcare costs with early physical therapy were an average $1011.22 lower (95% CI: 831.94-1190.50) than those with delayed physical therapy, with a mean cost of $8,687.25 (SE = 59.52) and $9,698.47 (SE = 69.46), respectively (Table 4).

**Table 3:** **Adjusted odds ratio with 99% confidence intervals of receiving specific utilization outcomes during the 2-year follow-up period based on the timing and adherence of physical therapy**

	**Adjusted OR**	**Confidence interval**
**Timing: Early vs. Delayed**		
Advanced imaging	0.52	0.50. 0.54
Spinal injection	0.56	0.53, 0.59
Lumbar surgery	0.59	0.54, 0.65
Opioid use	0.62	0.60, 0.64
**Content: Adherent vs. Non-Adherent**		
Advanced imaging	0.72	0.69, 0.76
Spinal injection	0.82	0.77, 0.87
Lumbar surgery	0.85	0.75, 0.96
Opioid use	0.97	0.93, 1.01

**Table 4:** **Utilization and costs outcomes occurring over the 2-year follow-up period for the entire sample and based on the combined categories of timing and adherence of physical therapy**

		**Early adherent (n = 17,175)**	**Early non-adherent (n = 23,993)**	**Delayed adherent (n = 13,742)**	**Delayed non-adherent (n = 16,649)**
**Utilization outcomes**					
Number of physical therapy visits (mean, sd)		6.3 (7.8)	15.8 (18.5)	6.0 (7.2)	13.9 (15.1)
Advanced imaging		12.8%	17.5%	22.2%	30.2%
Lumbar spinal injections		9.2%	11.1%	14.8%	17.6%
Lumbar spine surgery		2.1%	2.4%	3.3%	3.9%
Opioid medication use		60.4%	62.2%	71.1%	71.6%
**Costs incurred over the 24 month period** (mean, standard error)					
	Prescription medication cost	$905.79 (29.08)	$1,281.43 (28.61)	$862.57 (26.06)	$1,167.58 (25.88)
	Inpatient costs	$10,510.57 (324.10)	$10,547.58 (328.83)	$13,075.45 (541.37)	$12,352.16 (380.62)
	Total LBP costs	$1,914.26 (30.92)	$2,232.00 (29.62)	$3,067.57 (54.96)	$3,456.39 (49.43)
	Non‐LBP healthcare costs	$8,787.95 (112.26)	$8,866.12 (100.89)	$9,906.42 (145.07)	$9,576.34 (110.02)

### Healthcare utilization and costs based on guideline adherence

A determination of adherence vs. non-adherence among patients who utilized physical therapy could be made for 58.3% (n = 71,559) of patients, with 51,164 (41.7%) patients excluded from the determination of adherence because they were referred to physical therapy but only attended 1 physical therapy session and it is difficult to assess any pattern of care based on a single visit since the initial visit is predominantly limited to an evaluation. Among those, 43.2% (n = 30,917) were categorized as receiving care adherent to the recommendation for active treatment and 56.8% (n = 40,642) were categorized as non-adherent. Patients who received adherent care had a mean of 6.2 (sd = 7.6) physical therapy visits compared to 15.0 visits (sd = 17.2) for those receiving non-adherent care (Table 2).

Adjusting for the demographic and baseline co-morbid characteristics, the odds ratios for health services utilized during the 2-year follow-up period based on adherence to LBP practice guidelines are detailed in Table 3. Compared to those receiving non-adherent care, patients receiving adherent care were less likely to receive advanced imaging (AOR = 0.72, 99% CI: 0.69, 0.76), spinal injections (AOR = 0.82, 99% CI: 0.77, 0.87) and lumbar spine surgery (AOR = 0.85, 99% CI: 0.75, 0.96). There was no difference in opioid use based on adherence. Total LBP-related costs during the 2-year follow-up for patients receiving adherent care were an average $306.69 lower (95% CI: 227.63-385.75) compared to for patients receiving non-adherent care, with a mean cost of $2,426.88 (SE = 30.04) and $2,733.57 (SE = 26.92), respectively. Similarly, prescription medication costs with adherent care were an average $347.63 lower (95% CI: 292.63-402.63) than those with non-adherent care, with a mean cost of $886.27 (SE = 19.82) and $1233.90 (SE = 19.86), respectively (Table 4).

### Healthcare utilization and costs based on combined categories of timing referral and guideline adherence

**Figure 2:**
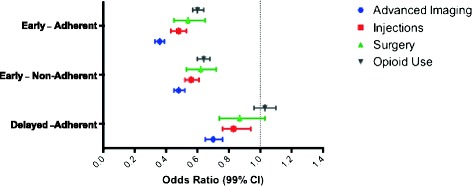
**Odds ratios for receiving specific utilization outcomes during the 2-year follow-up period based on the physical therapy timing and adherence combined categories, adjusting for all demographic and baseline co-morbid characteristics.** The reference category is delayed and non-adherent physical therapy.

Among the 71,559 patients who utilized physical therapy and for whom a determination of adherence could be made, 24.0% (n = 17,175) were categorized as receiving early physical therapy that was also adherent to the recommendation for active treatment, 19.2% (n = 13,742) received delayed physical therapy that was adherent, 23,993 (33.5%) received delayed and adherent care, and 16,649 (23.3%) received physical therapy that was delayed and non-adherent. Utilization of health services during the 2-year follow-up period based on both timing of care and adherence to guideline recommendations are detailed in Table 4. Compared to the sub-group receiving delayed and non-adherent physical therapy, patients receiving early and adherent physical therapy had a decreased likelihood of receiving advanced imaging (AOR = 0.36, 99% CI: 0.33, 0.39), spinal injections (AOR = 0.48, 99% CI: 0.43, 0.53), lumbar spine surgery (AOR = 0.54, 99% CI: 0.45, 0.65), or opioid use (AOR = 0.60, 99% CI: 0.57, 0.64). However, patients receiving delayed but adherent physical therapy were still less likely to receive injections or advanced imaging compared to the sub-group who received delayed and non-adherent care. The AORs and corresponding 95% CI are presented in Figure 2.

## Discussion

This study included a large sample of patients newly consulting a primary care physician for LBP in the MHS and extends our knowledge in several important ways on the implications of timing of care and adherence to practice guidelines for LBP. Unlike previous studies that have examined timing and adherence separately, [18,19] our large sample size examined the implications of both factors. Of particular interest, the 24% of patients who received early physical therapy that was also adherent to practice guidelines had the lowest utilization and costs compared to any of the other 3 possible combinations of timing and adherence. Early and adherent physical therapy was associated with significantly lower utilization of advanced imaging, lumbar spinal injections, lumbar spine surgery, and use of opioids. Given the enormous burden of excessive and unnecessary care for patients with LBP on society, cost savings from early guideline adherent physical therapy has important implications for single payer health care systems to design optimal care process models for LBP.

Compared to delayed and adherent physical therapy, patients receiving early and adherent physical therapy had 60% lower total LBP-related costs during the 2-year follow-up period (Table 4). Non-LBP healthcare and inpatient costs were also 13% and 24% lower, respectively, with early and adherent care, suggesting accretive health benefits from physical therapy that extend beyond the primary reason for seeking treatment (Table 4). In fact, when considering the various combinations, a dose–response relationship appears to exist in the sense that the results show a progressive increase in subsequent utilization and costs as care shifts from being classified as early and adherent compared to late and non-adherent care.

Although speculative, there are several possible explanations for why this may be the case. When following guideline based treatment, physical therapists provide patients with an active management approach that counter-balances management strategies that foster a sense of dependency in the patient [23]. For example, if physical therapists can assist in developing self-efficacy, it is reasonable to expect that the benefits of doing so would have the greatest impact when implemented early compared to late in the course of care, especially before negative expectations are reinforced and entrenched within the patient’s beliefs. This is in contrast to evidence that early use of advanced imaging, for example, reduces a patient’s sense of self efficacy and optimism for recovery, thus increasing the risk of subsequent procedures such as injection, surgery, and opioid medication [24,25]. For example, providing information on MRI results to patients with acute LBP has been shown to diminish a patient’s sense of well-being [26,27].

Although significant emphasis is being given to the importance of adherence to practice guidelines for costly conditions such as LBP, our results emphasize the importance of better aligning processes of care on two dimensions. However, there may be questions on the weighting of timing and guideline adherence in environments where both factors cannot be manipulated. In this case it seems that timing of referral may be more important. For example, recent studies involving Medicare [19] and commercial insurance samples [18] have demonstrated that early access to physical therapy regardless of adherence was strongly associated with a reduction in subsequent health care utilization and costs, evidenced by reduced risks of advanced imaging, surgery, spinal injections, and opioid use. This reduction in utilization translated into 67% lower total LBP-related costs during the 2-year follow-up (Table 2), reinforcing the notion that, given the inability to effect change in both timing and adherence, optimal timing for seeking care for LBP may overshadow the importance of guideline adherence, at least when studies have considered this factor in isolation. However, as results from this study indicated, there is additional benefit from guideline adherence, and the combination of these factors should be the goal for health care systems wanting to improve outcomes and decrease unwarranted utilization from LBP.

Our results extend the findings from civilian settings by demonstrating an association between early guideline adherent care and utilization and costs in a single payer health system. The MHS is a potential proxy for examining the implications of a single payer system in universal health care, thus the results are important to consider in light of the broader health care reform debate currently taking place in the United States. The fact that all patients had the same payer also mitigates potential differences in outcome based on level of insurance benefit and access to care, which is a common source of confounding in health economics research. Due to this environment, this study also has the advantage of being able to exclude financial incentives as having influenced referral to and utilization of physical therapy. Therefore, it is imperative to examine current guideline recommendations for managing LBP in light of these results.

Most clinical practice guidelines recommend only advice and education for all patients with non-specific LBP during the initial weeks of management, with consideration of psychosocial factors and referral to physical therapy recommended only when recovery is delayed. Psychosocial factors have been identified as risk factors that act as “obstacles to recovery” and increase the risk of developing chronic disability. Our study is unable to determine which patients benefit most from early referral versus those patients for whom self-management is adequate. It is likely that referring all patients with LBP is unnecessary and could increase overall costs. Recent research has demonstrated that targeted interventions to address the individual’s specific modifiable psychosocial prognostic indicators reduces disability, increases quality of life, and lowers health care costs [28]. Additionally, despite guideline recommendations to delay physical therapy, approximately 60% of patients who utilized physical therapy did so within the first few weeks after initial consultation with their primary care provider. A similar pattern has been reported in a commercial claims database in which 53% who went to physical therapy did so within 2 weeks after the primary care visit [18] and Medicare enrollees with a new consultation for LBP in which 75% received care within 4 weeks [19]. Given our current results and emerging evidence from multiple studies across federal and commercial payers that early access to a physical therapist is associated with significant reductions in subsequent health care utilization and overall costs of care, [18,19] recommendations in clinical practical guidelines to delay referral to physical therapy need to be re-examined.

The results of this study should be examined in light of the following limitations. Given the favorable natural history of LBP, many patients improve regardless of treatment. Those referred to physical therapy early are also more likely to have a shorter duration of pain, thus the potential for selection bias to have influenced these results. We accounted for a number of co-morbidities available in the data set and excluded patients with prior visits for LBP to mitigate against this possibility. However, the retrospective observational design of this study imposes limitations on extending the associations we observed to causation. Although we attempted to exclude patients with a specific spinal pathology, it is possible that a few patients may have been inadvertently included in the data set, in which case advanced imaging may be indicated. Additionally, although our results support that early physical therapy which adheres to practice guidelines may be less resource intense, we cannot conclude without patient-centered clinical outcomes (i.e., pain, function, disability, satisfaction, etc.) that the care was more cost effective. Further, it may be that the standard we used to judge adherence to practice guidelines (CPT codes) was not sufficiently sensitive to determine whether care is consistent with clinical practice guidelines. We also did not account for indirect or out-of-pocket costs for treatments such as complementary care, which is common for LBP [29]. However, it is likely that the observed effects on total costs would have been even larger had these costs been considered.

## Conclusion

Initial management decisions following a new episode of LBP have profound implications for clinical outcomes and downstream utilization and costs. In this study early referral to guideline adherent physical therapy was associated with lower utilization of advanced imaging, lumbar spinal injections, lumbar spine surgery, and use of opioids. Cost savings from early guideline adherent physical therapy has important implications for designing optimal care process models in single payer systems. Future research is necessary to examine which patients with LBP benefit early physical therapy and determine strategies for providing early guideline adherent care. Randomized controlled studies are also needed to evaluate whether such observational findings are causative before definitive policy recommendations can be made.

## Additional file

Additional file 1: Table S1. ICD-9 codes used to identify low back pain. **Table S2.** ICD-9 codes used to identify non-musculoskeletal reasons for low back pain.

## References

[1] Ma VY, Chan L, Carruthers KJ (2014). Incidence, prevalence, costs, and impact on disability of common conditions requiring rehabilitation in the United States: stroke, spinal cord injury, traumatic brain injury, multiple sclerosis, osteoarthritis, rheumatoid arthritis, limb loss, and back pain. Arch Phys Med Rehabil.

[2] Martin BI, Deyo RA, Mirza SK, Turner JA, Comstock BA, Hollingworth W (2008). Expenditures and health status among adults with back and neck problems. JAMA.

[3] Mafi JN, McCarthy EP, Davis RB, Landon BE (2013). Worsening trends in the management and treatment of back pain. JAMA Intern Med.

[4] Smith M, Davis MA, Stano M, Whedon JM (2013). Aging baby boomers and the rising cost of chronic back pain: secular trend analysis of longitudinal Medical Expenditures Panel Survey data for years 2000 to 2007. J Manipulative Physiol Ther.

[5] Meucci RD, Fassa AG, Paniz VM, Silva MC, Wegman DH (2013). Increase of chronic low back pain prevalence in a medium-sized city of southern Brazil. BMC Musculoskelet Disord.

[6] Knox J, Orchowski J, Scher DL, Owens BD, Burks R, Belmont PJ (2011). The incidence of low back pain in active duty United States military service members. Spine (Phila Pa 1976).

[7] Cohen SP, Gallagher RM, Davis SA, Griffith SR, Carragee EJ (2012). Spine-area pain in military personnel: a review of epidemiology, etiology, diagnosis, and treatment. Spine J.

[8] Cohen SP, Nguyen C, Kapoor SG, Anderson-Barnes VC, Foster L, Shields C (2009). Back pain during war: an analysis of factors affecting outcome. Arch Intern Med.

[9] Brooks DE, Agochukwu UF, Arrington ED, Mok JM (2013). Psychological distress in the active duty military spine patient. Mil Med.

[10] Golub A, Bennett AS (2013). Prescription opioid initiation, correlates, and consequences among a sample of OEF/OIF military personnel. Subst Use Misuse.

[11] General OTAS (2010). Pain Management Task Force - Final Report.

[12] Chou R, Huffman LH, American Pain S (2007). American College of P. Nonpharmacologic therapies for acute and chronic low back pain: a review of the evidence for an American Pain Society/American College of Physicians clinical practice guideline. Ann Intern Med.

[13] Chou R, Huffman LH, American Pain S (2007). American College of P. Medications for acute and chronic low back pain: a review of the evidence for an American Pain Society/American College of Physicians clinical practice guideline. Ann Intern Med.

[14] Fourney DR, Dettori JR, Hall H, Hartl R, McGirt MJ, Daubs MD (2011). A systematic review of clinical pathways for lower back pain and introduction of the Saskatchewan Spine Pathway. Spine (Phila Pa 1976).

[15] Koes BW, van Tulder M, Lin CW, Macedo LG, McAuley J, Maher C (2010). An updated overview of clinical guidelines for the management of non-specific low back pain in primary care. Eur Spine J.

[16] Savigny P, Watson P, Underwood M, Guideline DG (2009). Early management of persistent non-specific low back pain: summary of NICE guidance. BMJ.

[17] Williams CM, Maher CG, Hancock MJ, McAuley JH, McLachlan AJ, Britt H (2010). Low back pain and best practice care: A survey of general practice physicians. Arch Intern Med.

[18] Fritz JM, Childs JD, Wainner RS, Flynn TW (2012). Primary care referral of patients with low back pain to physical therapy: impact on future health care utilization and costs. Spine (Phila Pa 1976).

[19] Gellhorn AC, Chan L, Martin B, Friedly J (2012). Management patterns in acute low back pain: the role of physical therapy. Spine (Phila Pa 1976).

[20] Mundell BF, Friedberg MW, Eibner C, Mundell WC (2013). US military primary care: problems, solutions, and implications for civilian medicine. Health Aff (Millwood).

[21] Moses H, Matheson DH, Dorsey ER, George BP, Sadoff D, Yoshimura S (2013). The anatomy of health care in the United States. JAMA.

[22] Childs JD, Fritz JM, Flynn TW, Irrgang JJ, Johnson KK, Majkowski GR (2004). A clinical prediction rule to identify patients with low back pain most likely to benefit from spinal manipulation: a validation study. Ann Intern Med.

[23] Costa Lda C, Maher CG, McAuley JH, Hancock MJ, Smeets RJ (2011). Self-efficacy is more important than fear of movement in mediating the relationship between pain and disability in chronic low back pain. Eur J Pain.

[24] Webster BS, Cifuentes M (2010). Relationship of early magnetic resonance imaging for work-related acute low back pain with disability and medical utilization outcomes. J Occup Environ Med.

[25] Franklin GM, Stover BD, Turner JA, Fulton-Kehoe D, Wickizer TM (2008). Disability Risk Identification Study C. Early opioid prescription and subsequent disability among workers with back injuries: the Disability Risk Identification Study Cohort. Spine (Phila Pa 1976).

[26] Ash LM, Modic MT, Obuchowski NA, Ross JS, Brant-Zawadzki MN, Grooff PN (2008). Effects of diagnostic information, per se, on patient outcomes in acute radiculopathy and low back pain. AJNR Am J Neuroradiol.

[27] Modic MT, Obuchowski NA, Ross JS, Brant-Zawadzki MN, Grooff PN, Mazanec DJ (2005). Acute low back pain and radiculopathy: MR imaging findings and their prognostic role and effect on outcome. Radiology.

[28] Hill JC, Whitehurst DG, Lewis M, Bryan S, Dunn KM, Foster NE (2011). Comparison of stratified primary care management for low back pain with current best practice (STarT Back): a randomised controlled trial. Lancet.

[29] Santaguida PL, Gross A, Busse J, Gagnier J, Walker K, Bhandari M (2009). Complementary and alternative medicine in back pain utilization report. Evid Rep Technol Assess (Full Rep).

