# Erratum to: Implications of early and guideline adherent physical therapy for low back pain on utilization and costs

**DOI:** 10.1186/s12913-016-1681-2

**Published:** 2016-08-26

**Authors:** John D. Childs, Julie M. Fritz, Samuel S. Wu, Timothy W. Flynn, Robert S. Wainner, Eric K. Robertson, Forest S. Kim, Steven Z. George

**Affiliations:** 1 Army Medical Department Center and School, US Army-Baylor University Doctoral Program in Physical Therapy, 3151 Scott Rd., Rm. 2307, JBSA Fort Sam Houston, San Antonio, TX 78234 USA; 2 Department of Physical Therapy, University of Utah, 520 Wakara Way, Salt Lake City, UT 84108 USA; 3 Department of Health Services Research, Management and Policy, College of Public Health and Health Professions, University of Florida, 1329 SW 16th St., Rm. 5231, Gainesville, FL 32610-0177 USA; 4 EIM School of Physical Therapy, South College, 3904 Lonas Dr, Knoxville, TN 37909 USA; 5 Doctor of Physical Therapy Program, University of Texas at El Paso, 500 W. University Avenue, El Paso, TX 79968 USA; 6 US Army Medical Department Center and School, US Army-Baylor MHA/MBA Program, 3599 Winfield Scott Rd., Bldg. 2841, JBSA Fort Sam Houston, San Antonio, TX 78234-6135 USA; 7 Department of Physical Therapy, Director, Brooks-PHHP Research Collaboration, University of Florida, P.O. Box 100154, Gainesville, FL 32610-0154 USA

## Erratum

There is a typographical error on p. 7 of the article [1], where it states, “…24.0 % (*n* = 17,175) were categorized as receiving early physical therapy that was also adherent to the recommendation for active treatment, 19.2 % (*n* = 13,742) received **delayed** physical therapy that was **adherent**, 23,993 (33.5 %) received **delayed** and **adherent** care, and 16,649 (23.3 %) received physical therapy that was delayed and non-adherent.” As can be seen, reference to the “delayed and adherent” category is mentioned twice.

Based on Table 4, the second reference to “delayed and adherent” care should have read “early and non-adherent” care. The corrected text is as follows:

“…24.0 % (*n* = 17,175) were categorized as receiving early physical therapy that was also adherent to the recommendation for active treatment, 19.2 % (*n* = 13,742) received delayed physical therapy that was adherent, 23,993 (33.5 %) received **early** and **non-adherent** care, and 16,649 (23.3 %) received physical therapy that was delayed and non-adherent.”

This typographical correction makes the text in the manuscript now consistent with the data in Table 4, which is correct as originally published.

## References

[1] Childs et al. Implications of early and guideline adherent physical therapy for low back pain on utilization and costs. BMC Health Serv Res. 2015; 15:150. doi: 10.1186/s12913-015-0830-3 10.1186/s12913-015-0830-3 PMC4393575 25880898

