# Supplementary material for: Implications of early and guideline adherent physical therapy for low back pain on utilization and costs
Source: BMC Health Serv Res. 2015 Apr 9;15:150. doi: 10.1186/s12913-015-0830-3 (PMC4393575; doi:10.1186/s12913-015-0830-3)
Supplement: Additional file 1: Table S1. — ICD-9 codes used to identify low back pain. Table S2. ICD-9 codes used to identify non-musculoskeletal reasons for low back pain. [file 12913_2015_830_MOESM1_ESM.docx]

**Additional file 1**

**Table S1.** ICD-9 codes used to identify low back pain

| **Code** | **Description** | **Code** | **Description** |
| --- | --- | --- | --- |
| 721.3 | Lumbosacral spondylosis without myelopathy | 724.5 | Backache, unspecified |
| 722.1 | Lumbar disc displacement | 756.11 | Spondylolysis, lumbosacral region |
| 722.52 | Lumbar/ lumbosacral disc displacement | 756.12 | Spondylolesthesis |
| 722.73 | Lumbar disc disease with myelopathy | 846.0 | Sprain - lumbosacral |
| 722.93 | Other disc disorder – lumbar region | 846.1 | Sprain - sacroiliac |
| 724.02 | Spinal stenosis-lumbar | 846.8 | Sprain – other specified sites of sacroiliac region |
| 724.2 | Lumbago | 846.9 | Sprain – unspecified site of sacroiliac region |
| 724.3 | Sciatica | 847.2 | Sprain – lumbar region |
| 724.4 | Thoracic or lumbosacral neuritis or radiculitis, unspecified | 847.3 | Sprain – sacrum |

**Table S2.** ICD-9 codes used to identify non-musculoskeletal reasons for low back pain

| **ICD-9 code** | **Description** |
| --- | --- |
| 592.xx | Calculus of kidney |
| 574.2 | Calculus of gallbladder without mention of cholecystitis |
| 599.0 | Urinary tract infection, site not specified |
| V13.02 | Urinary (tract) infection |
| 140.xx – 239.xx | Neoplasms |
| V17.81, V82.81 | Osteoporosis |
| 344.6 | Cauda equine syndrome |
| 730.xx | Osteomyelitis, periostitis, and other infections involving bone |
| 731.3 | Major osseous deficit |

| **Code** | **Description** |
| --- | --- |
| 00630 | Anesthesia for procedures in lumbar region; not otherwise specified. |
| 00670 | Anesthesia for extensive spine and spinal cord procedures. |
| 20930 | Allograft for spine surgery only; morselized. |
| 20936 | Autograft for spine surgery only; local, obtained from same incision. |
| 22102 | Partial excision of posterior vertebral component for intrinsic bony lesion, single segment; lumbar. |
| 22103 | Partial excision of posterior vertebral component for intrinsic bony lesion, single segment; each additional segment. |
| 22224 | Osteotomy of spine, including diskectomy, anterior approach, single segment; lumbar. |
| 22226 | Osteotomy of spine, including diskectomy, anterior approach, single segment; each additional segment. |
| 22558 | Arthrodesis, anterior interbody technique, including minimal diskectomy to prepare interspace; lumbar. |
| 22585 | Arthrodesis, anterior interbody technique, including minimal diskectomy to prepare interspace; each additional interspace. |
| 22612 | Arthrodesis, posterior or posterolateral technique, single level; lumbar |
| 22630 | Arthrodesis, posterior interbody technique, single interspace; lumbar |
| 22802 | Arthrodesis, posterior, for spinal deformity, with or without cast; 7 to 12 vertebral segments. |
| 22840 | Insert spine fixation device |
| 22842 – 22844 | Posterior segmental instrumentation; 3 – 6 vertebral segments thru 13 or more segments |
| 22851 | Apply spine prosthetic device |
| 62287 | Aspiration procedure, percutaneous, of nucleus pulposus of intervertebral disk, any method, single or multiple levels, lumbar. |
| 63005 | Laminectomy with exploration and/or decompression of spinal cord and/or cauda equine, without facetectomy, foraminotomy or diskectomy, (eg, spinal stenosis), one or two vertebral segments; lumbar, except for spondylolisthesis. |
| 63011, 63012 | Laminectomy with exploration and/or decompression of spinal cord and/or cauda equine, without facetectomy, foraminotomy or diskectomy, one or two vertebral segments; sacral or lumbar |
| 63030 | Laminotomy with decompression of nerve root(s), including partial facetectomy, foraminotomy and/or excision of herniated intervertebral disk; one interspace, lumbar. |
| 63035 | Laminotomy with decompression of nerve root(s), including partial facetectomy, foraminotomy and/or excision of herniated intervertebral disk; each additional interspace, cervical or lumbar. |
| 63042 | Laminotomy with decompression of nerve root(s), including partial facetectomy, foraminotomy and/or excision of herniated intervertebral disk, re-exploration; lumbar. |
| 63047 | Laminectomy, facetectomy and foraminotomy (unilateral or bilateral with decompression of spinal cord, cauda quine and/or nerve root(s)) , single vertebral segment; lumbar. |
| 63048 | Removal of spine lamina – add-on |
| 63088 – 63091 | Vertebral corpectomy, partial or complete, combined thoracolumbar approach with decompression of spinal cord, cauda equine or nerve root(s), lower thoracic or lumbar; each additional segment. |
| 63185, 63190 | Laminectomy with rhizotomy |
| 63200 | Laminectomy, with release of tethered spinal cord, lumbar. |
| 63267, 63272 | Laminectomy for excision or evacuation of intraspinal lesion other than neoplasm, extradural or intradural; lumbar. |
| 63290 | Laminectomy for biopsy/excision of intraspinal neoplasm; combined extra-intradural lesion, any level. |
| 63303 | Vertebral corpectomy, partial or complete, for excision of intraspinal lesion, single segment; extradural, lumbar or sacral by transperitoneal or retroperitoneal approach. |
| 63047 | Laminectomy, facetectomy and foraminotomy (unilateral or bilateral with decompression of spinal cord, cauda equine and/or nerve root(s)) , single vertebral segment; lumbar. |
| 63048 | Removal of spine lamina – add-on |
| 64622 | Destruction by neurolytic agent; paravertebral facet joint nerve, lumbar, single level. |
| 64623 | Destruction by neurolytic agent; paravertebral facet joint nerve, lumbar, each additional level. |

**Table 3.** CPT-4 codes used to identify prior surgery for low back pain

| **Code** | **Description** |
| --- | --- |
| CO-MORBID MENTAL HEALTH CONDITIONS | |
| 296.xx | Affective psychoses |
| 297.xx | Delusional disorders |
| 298.xx | Other nonorganic psychoses |
| 300.xx | Neurotic disorders |
| 301.xx | Personality disorders |
| 308.xx | Acute reaction to stress |
| 309.xx | Adjustment reaction |
| 311.xx | Depressive disorders, not elsewhere classified |
| CO-MORBID NECK / THORACIC PAIN: | |
| 721.0 | Cervical spondylosis without myelopathy |
| 721.1 | Cervical spondylosis with myelopathy |
| 721.2 | Thoracic spondylosis without myelopathy |
| 721.41 | Spondylogenic compression of thoracic spinal cord |
| 722.0 | Displacement of cervical intervertebral disc without myelopathy |
| 722.4 | Degeneration of cervical intervertebral disc |
| 722.51 | Intervertebral disc disorder, thoracic or thoracolumbar region |
| 722.71 | intervertebral disc disorder without myelopathy – cervical |
| 722.72 | Intervertebral disc disorder without myelopathy, thoracic region |
| 722.81 | Post-laminectomy syndrome - cervical |
| 722.91 | other unspecified disc disorder – cervical region |
| 723.xx | Other disorders of cervical region |
| 724.1 | Pain in thoracic spine |
| 739.1 | Non-allopathic lesions not otherwise specified – cervical region |
| 805 | Fracture of cervical spine without spinal cord injury |
| 847.0 | Sprains and strains of other, unspecified part of the back - neck |
| 953.0 | Injury to nerve root and spinal plexus - cervical |
| 954.0 | Injury to other nerves of trunk - cervical |
| CO-MORBID FIBROMYALGIA | |
| 729.1 | Myalgia and myositis, unspecified |

**Table 4.** ICD-9 codes used to identify co-morbid conditions
